# The role of H19, a long non-coding RNA, in mouse liver postnatal maturation

**DOI:** 10.1371/journal.pone.0187557

**Published:** 2017-11-03

**Authors:** Chad Pope, Stephanie C. Piekos, Liming Chen, Shashank Mishra, Xiao-bo Zhong

**Affiliations:** 1 Department of Pharmaceutical Sciences, School of Pharmacy, University of Connecticut, Storrs, Connecticut, United States of America; 2 Department of Physiology and Neurobiology, University of Connecticut, Storrs, Connecticut, United States of America; Digestive Disease Research Center, Scott & White Healthcare, UNITED STATES

## Abstract

H19 RNA is highly expressed at early postnatal ages and precipitously decreases at a specific time corresponding with increases in expression of genes important for mature liver function, such as drug metabolizing enzymes. H19’s role in the regulation of liver maturation is currently unknown. Using an *H19* knockout mouse model to determine the role of H19 in liver development, we quantified gene expression for insulin growth factor signaling, Wnt signaling, key cytochrome P450 (P450) enzymes known to change as the liver develops, and fetal and adult plasma protein produced in liver. In mice lacking H19 expression, liver weights were significantly increased immediately after birth and significant increases were found in the number of actively proliferating cells. Increases in cell proliferation may be due to increases in β-catenin protein affecting Wnt signaling, increases in insulin-like growth factor 2 (IGF2) expression, and/or increases in insulin-like growth factor 1 receptor (IGF1R) expression at the protein level. Loss of targeted inhibition of IGF1R by microRNA 675 (miR-675) may be the cause of IGF1R increases, as miR-675 expression is also abrogated with loss of H19 expression in our model. P450 expression patterns were largely unchanged. No change in the production of plasma proteins was found, indicating H19 may not be important for liver maturation despite its role in controlling cell proliferation during liver growth. H19 may be important for normal liver development, and understanding how the liver matures will assist in predicting drug efficacy and toxicity in pediatric populations.

## Introduction

Liver development requires both cell proliferation and differentiation. Cell proliferation allows the liver to achieve its proper size in the body and cell differentiation allows the liver to attain proper functions. Both events coincide concurrently during postnatal liver maturation. During this important, but understudied, phase of development, the liver undergoes a switch in functions. In mouse, the liver becomes the major hematopoietic organ in the fetus between embryonic days 10 to 15 [1]. After birth it matures into an organ primarily functioning in metabolism [2]. Throughout ontogenesis until the liver is fully mature, changes occur in the expression profiles of many protein-coding genes involved in important liver functions [3,4], including the regulation of energy metabolism [5] and drug metabolism and transport [6–10], which are responsible for important physiological functions of mature liver. However, it is not fully understood what initiates this switch in functions.

Proteins involved in signaling pathways and regulation of splicing are implicated in playing important roles in cell growth and differentiation during postnatal liver maturation. Knockout of mouse liver β-catenin, the intracellular transducer in Wnt signaling, results in a decrease in cell proliferation and a decrease in postnatal liver size [11]. Yes-associated protein, the downstream effector of Hippo Kinase signaling, affects cell proliferation and regulates genes controlling hepatic functions including metabolism of bile acids and retinoic acids in mouse [12]. Two splicing factors are also known to affect postnatal liver maturation. Epithelial splicing regulatory protein 2 is induced in hepatocytes during the postnatal age and controls the neonatal-to-adult switch of splice isoforms in gene transcripts involved in proliferation and differentiation that control hepatocyte maturation in both mouse and human [13]. Serine/arginine-rich splicing factor 3 (SRSF3) alters splicing of genes that regulate glucose and lipid metabolism. Knockout of *Srsf3* in mice results in a decline in postnatal liver growth by a prolonged expression of fetal markers including persistence of hepatic hematopoiesis [14]. Clearly, protein-coding genes are important for the functional switch in developing liver, however, the role of non-coding genes has largely been unexplored.

Long non-coding RNAs (lncRNAs) also change in identifiable expression patterns throughout ontogenesis and potentially regulate liver development. Recently, our laboratory examined the alterations of gene expression in lncRNAs during the liver’s functional transition through the prenatal stage to adult life in mice [15]. Three major ontogenic expression patterns were found with specific lncRNAs enriched at the neonatal, adolescent, and adult ages, indicating lncRNAs are developmentally regulated. Expression of lncRNAs with their neighboring protein-coding genes was found to be correlated, indicating lncRNAs may regulate the expression of nearby protein-coding genes or share regulatory regions within their gene loci that coordinate their expression. LncRNA H19 was found to be most differentially expressed lncRNA comparing prenatal expression to adult expression, and its *cis* protein-coding partner, IGF2, an important fetal growth factor, exhibits a similar temporal expression pattern. Despite being one of the most studied lncRNAs, H19’s role in liver development has not been fully determined.

H19 is involved in normal liver physiology and is implicated in liver diseases [16]. H19 is expressed in liver during periods of increased cell proliferation, including liver regeneration after injury [17] and hepatocellular carcinoma [18–20]. H19 is also highly expressed in fetal liver during organogenesis. In a human fetal liver cell line, H19 expression inhibits cell proliferation through Wnt signaling potentially to prevent overgrowth of fetal liver tissue [21]. We aim to test whether H19 plays a similar role in postnatal liver development, including both liver growth and liver maturation, using an *H19* knockout mouse model [22].

To test the effect of H19 on postnatal liver development, we used three genetically different mouse groups. *H19* is an imprinted gene uniquely expressed only from the maternal allele [23]. Therefore, we carefully bred *H19* knockout mice to generate mice heterozygous for the null mutation strictly controlling which parental allele *H19* is fully intact or fully removed. Using wild type mice (*H19*^+/+^), *H19* maternal allele knockouts (*H19*^M-/P+^), and *H19* paternal allele knockouts (*H19*^M+/P-^), we were able to assess the role of H19 in postnatal liver development when H19 is expressed or not expressed from a specific allele.

## Materials and methods

### Animals

**Fig 1:**
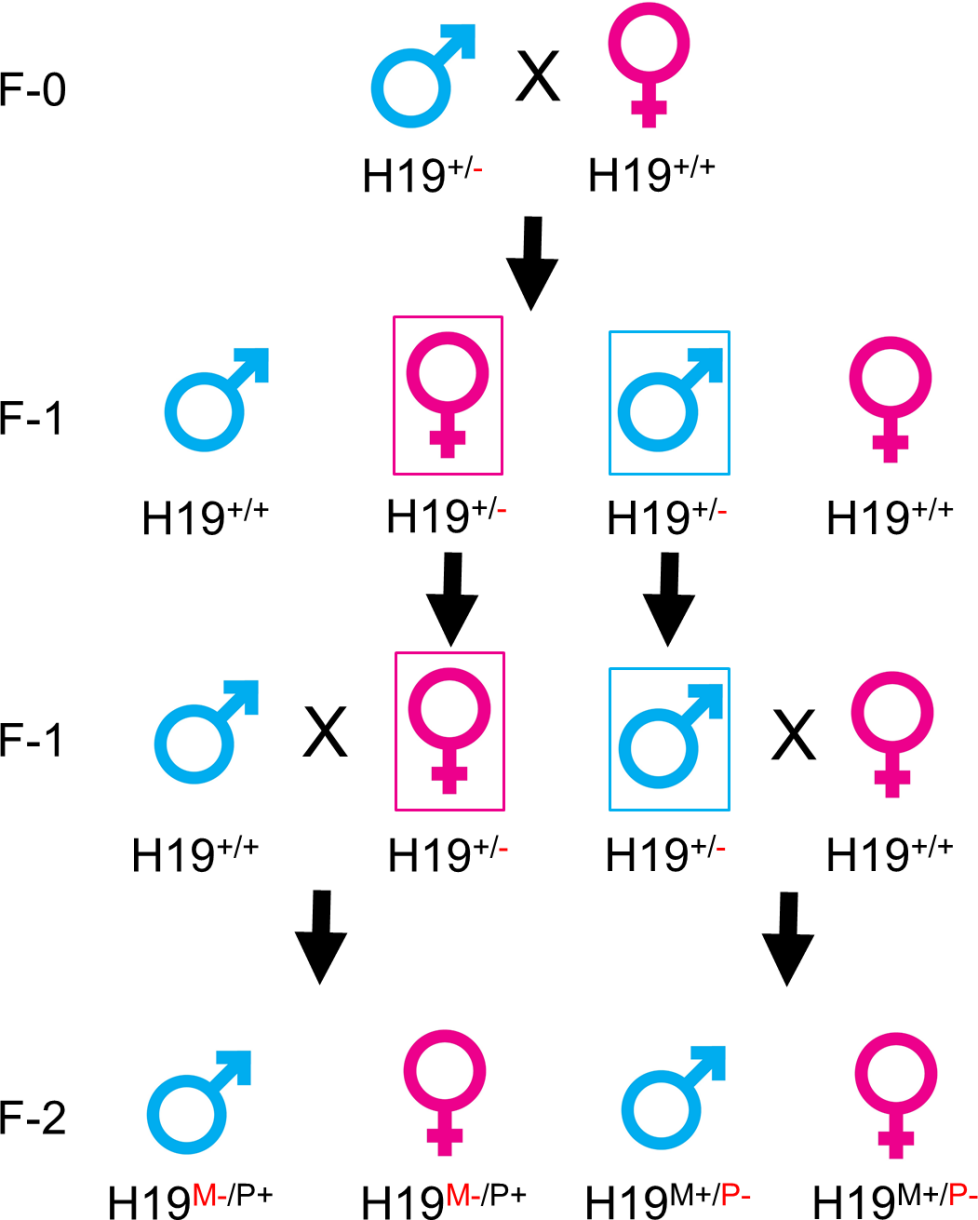
Breeding scheme to generate *H19* heterozygous paternal and maternal allele knockout mice.

Animals used to generate preliminary RNA-sequencing (RNA-Seq) data were previously described [15]. In this study, eight-week-old C57BL/6 mouse breeding pairs were purchased from Jackson Laboratory (Bar Harbor, ME). A permission for the use of previously characterized *H19* knockout mice [22] was received from Dr. Luisa Dandolo. A pair of *H19* heterozygous male knockout mice (male-*H19*^+/-^) were received from Dr. Linheng Li’s laboratory at the Stowers Institute for Medical Research (Kansas City, MO, USA). Mice were housed according to the animal care guidelines provided by the American Association for Animal Laboratory Sciences and were bred under standard conditions in the Laboratory Animal Resources Facility at the University of Connecticut (Protocol Number: A15-040). The use of these mice was approved by the Institutional Animal Care and Use Committee, Office of Research Compliance. A breeding scheme to generate paternal and maternal *H19* knockout mice is illustrated in Fig 1. Male heterozygous *H19* knockout (*H19*^+/-^) mice (F-0) carrying the *H19*^-^ on one allele with undetermined parental origin were initially bred with wild type (*H19*^+/+^) mice of the same C57BL/6 background to generate F-1 male-*H19*^+/-^ and female-*H19*^+/-^ heterozygous. The F-1 female *H19*^+/-^ were further bred with wild type mice to generate F-2 male and female maternal *H19* knockout (*H19*^M-/P+^) offspring and the F-1 male-*H19*^+/-^ were further bred with wild type mice to generate F-2 male and female paternal *H19* knockout (*H19*^M+/P-^) offspring. F-2 wild type (*H19*^M+/P+^) mice generated were used as controls. Liver samples were collected at the following ages: day 5, 10 (neonatal), 15, 20, 30 (adolescent), and 60 (adult) after birth. Livers from both males and females were collected for days 30 and 60 only, and livers from animals collected before age 30 days were not considered sexually mature and not separated by sex. The livers were immediately frozen in liquid nitrogen and stored at -80°C or fixed in formalin.

### Genotyping

Ear or tail snips were collected from mice for DNA extraction. PCR reactions were performed for identification of H19 deletion using a REDExtract-N-Amp Tissue PCR Kit purchased from Sigma-Aldrich (St. Louis, MO, USA). Primers were designed using a PrimerQuest Tool from Integrated DNA Technologies (Coralville, IA, USA). Sequences for the primers are forward 5’-CTGTTCATACTCCGTGGGATAG-3’, forward CAGACATTCATCCCGGTTACTT-3’, reverse 5’-CCTACCCATTACGAGCCTTAC-3’, and reverse 5’-GGGACCCATCTGTGTCTTGT-3’.

### Reverse transcription polymerase chain reaction (RT-PCR)

Total RNAs were isolated from livers without gallbladders using TRIzol reagent from Life Technologies (Guilford, CT, USA) according to the manufacturer's protocol. RNA concentrations were determined using a Nano Drop spectrophotometer from Nano Drop Technologies (Wilmington, DE, USA) at a wavelength of 260 nm. The integrity of the total RNAs was evaluated on an Agilent 2200 Tape Station from Agilent Technologies (Santa Clara, CA, USA). Gene expression at the RNA level of H19, miR-675-3p, miR-675-5p, IGF2, IGF1, IGF1R, β-catenin, cyclin D1, CYP3A16, CYP3A11, CYP2B10, CYP2C29, and GAPDH was determined by TaqMan assays from Life Technologies (Carlsbad, CA, USA) according to the manufacturer's protocol. Data were analyzed by generation of cycling time (Ct) and delta Ct (ΔCt) values for all genes against GAPDH.

### Immunohistochemistry

Fresh liver tissues were fixed in 10% buffered formalin, embedded in paraffin, and sections were prepared and stained by the Connecticut Veterinary Medical Diagnostic Laboratory at the University of Connecticut (Storrs, CT, USA). Immunohistochemistry was performed using antibodies against Ki-67 (rabbit polyclonal, Cat. No. ab15580) and proliferating cell nuclear antigen (PCNA) (rabbit polyclonal, Cat. No. ab18197) from Abcam (Cambridge, UK). A secondary antibody conjugated to peroxidase allowed for color precipitation using a VECTOR NovaRED Peroxidase (HRP) Substrate Kit from Vector Laboratories (Burlingame, CA, USA). Images were captured using an EVOS XL Core Cell Imaging System from Thermo Fisher Scientific (Waltham, MA, USA). Positively stained nuclei were identified by using the ImageJ image processing program, version 1.50i, from National Institutes of Health (Bethesda, MD, USA) using a color brightness threshold with a signal intensity greater than or equal to 200.

### Western blotting

Total proteins in liver lysates were isolated in RIPA Buffer and protein concentrations were determined by using a Qubit 2.0 Fluorometer by Invitrogen (Carlsbad, CA, USA) and the Lowry protein assay from Bio-Rad (Hercules, CA, USA) with absorbance at 750 nm using a Spectra MAX 190 from Molecular Devices (Sunnyvale, CA, USA). Proteins were run on polyacrylamide gels using the Mini-PROTEAN Tetra System by Bio-Rad (Hercules, CA, USA) and transferred onto PVDF membranes. Primary antibodies purchased from Cell Signaling Technology (Danvers, MA, USA) include IGF1R (1:1000, rabbit polyclonal, Cat. No. 3027), active β-catenin (1:1000, rabbit monoclonal, Cat. No. 8814), and total β-catenin (1:1000, rabbit monoclonal, Cat. No. 8480), and albumin (1:1000, rabbit polyclonal, Cat. No. 4929). Primary antibodies against Wnt6 (1:1000, rabbit polyclonal, Cat. No. ab50030) were purchased from Abcam (Cambridge, UK), α-fetoprotein (1:100, mouse monoclonal, Cat. No. MA5-12754) from Thermo Fisher Scientific (Waltham, MA, USA), and GAPDH (1:3000, rabbit polyclonal, Cat. No. G9545) from Sigma-Aldrich (St. Louis, MO, USA). Secondary antibodies, anti-rabbit IgG (1:1000, goat, Cat. No. 7074) and anti-mouse IgG (1:2000, horse, Cat. No. 7076), were purchased from Cell Signaling Technology (Danvers, MA, USA). Primary antibodies were conjugated to secondary antibodies conjugated to Horseradish peroxidase for detection using a ChemiDoc MP Imaging System from Bio-Rad (Hercules, CA, USA).

### Statistical analysis

The data are shown as the mean ± standard deviation. The significance of differences between means was determined using two-tailed unpaired Student’s *t* tests when two groups were compared. ANOVA was used when means were compared between more than two groups. The Kruskal-Wallis test was used when data were not normally distributed. Statistical analyses were performed using Prism7, version 7.01 from GraphPad Software, Inc. (La Jolla, CA, USA). Differences were considered to be significant if *p*<0.05.

## Results

### Ontogenic expression of H19 in liver during postnatal maturation

**Fig 2:**
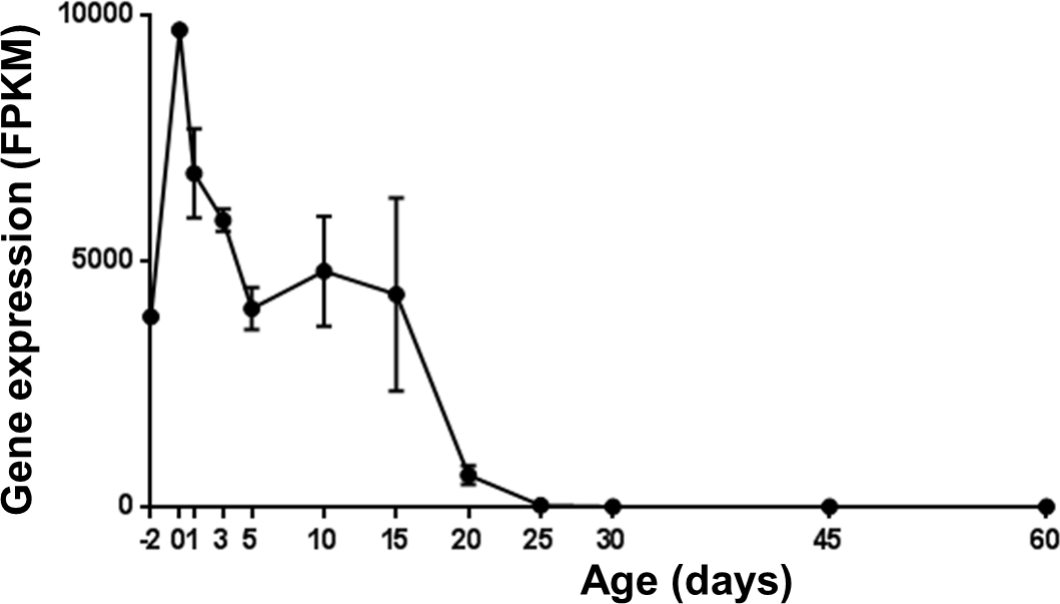
Ontogenic expression of H19 in mice during postnatal maturation in liver. Expression of H19 was determined by RNA-Seq in mouse livers at ages -2, 0, 3, 5, 10, 15, 20, 30, 45, and 60 days after birth (n = 3 per group). H19 expression level was measured as FPKM (Fragments Per Kilobase of transcript per Million mapped reads). Values are represented as mean ± S.D.

Previously generated RNA-Seq data [15] were used to examine H19 expression in wild type mouse liver (n = 36) at fetal, postnatal, and adult ages (Fig 2). In mouse liver, H19 is highly expressed before birth and rises to its highest level of expression around the time of birth. Expression levels precipitously decline at a postnatal age (approximately 20 days after birth) to nearly undetectable levels. Adult mouse liver does not express H19.

### Abolishment of expression of H19 and miR-675 in *H19* knockout mice

**Fig 3:**
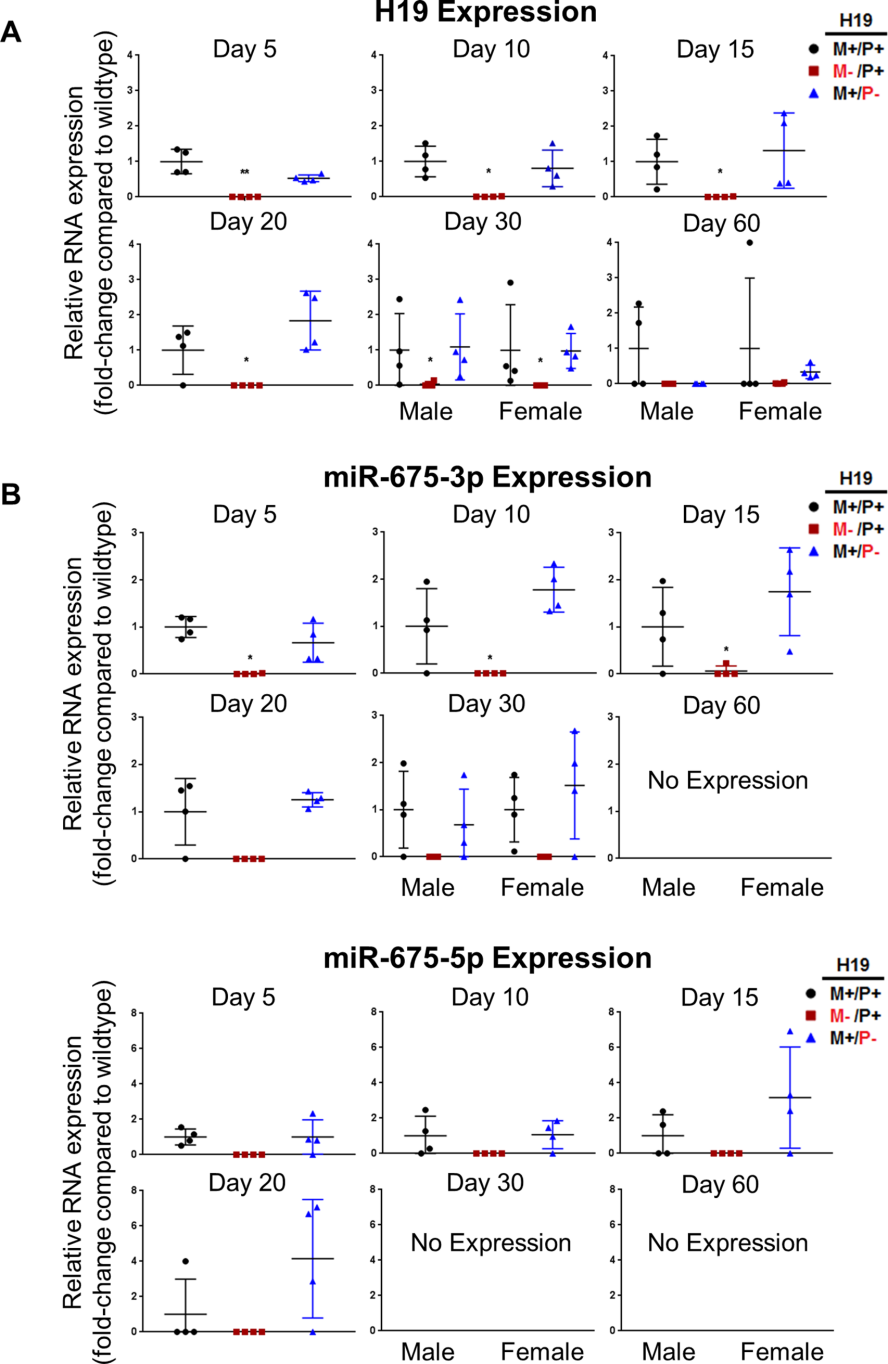
Expression of H19 and miR-675 RNA in mouse livers with *H19* knockout on different parental alleles. RNA expression of H19 (A) and miR-675-3p and miR-675-5p (B) in mouse livers at ages 5, 10, 15, 20, 30, and 60 days after birth (n = 4 per group) was determined by RT-PCR in wild type (*H19*^M+/P+^), maternal *H19* knockout (*H19*^M-/P+^), and paternal *H19* knockout (*H19*^M+/P-^) mice measured as fold-changes compared to the wild type. Values are represented as mean ± S.D. * *p*<0.05, ** *p*<0.01, *** *p*<0.001.

Expression of H19 in livers of wild type, maternal *H19* knockout, and paternal *H19* knockout mice was determined by RT-PCR at ages of 5, 10, 15, 20, 30, and 60 days after birth (Fig 3A). In all ages examined, H19 was not expressed in mice when the gene knockout was on the maternal allele despite the mice possessing an intact paternal allele (*H19*^M-/P+^). Wild type mice (*H19*^+/+^) and heterozygous mice with *H19* knockout on the paternal allele (*H19*^M+/P-^) exhibited similar expression of H19 at all ages.

Two different conserved microRNAs, miR-675-3p and miR-675-5p, are produced from the first exon of *H19* [24]. Using two different sets of primers directed against each miR-675 variant, their expression was determined by RT-PCR (Fig 3B). The pattern of means for both miR-675-3p and miR-675-5p expression followed H19 expression. In most wild type individuals, expression was high at early ages until approximately 20 days after birth when levels precipitously decline. Certain wild type individuals, despite normally expressing H19 at early ages, showed very little miR-675 expression, indicating large interindividual variation. Similar to H19, miR-675 was only expressed when *H19* was intact at the maternal allele (*H19*^M+/P+^ and *H19*^M+/P-^) while knockout on the paternal allele (*H19*^M+/P-^) was inconsequential to miR-675 expression. Essentially, *H19* maternal allele knockout mice (*H19*^M-/P+^) are also miR-675 knockout mice.

### Changes of liver and body weights in the absence of H19 expression during postnatal maturation

**Fig 4:**
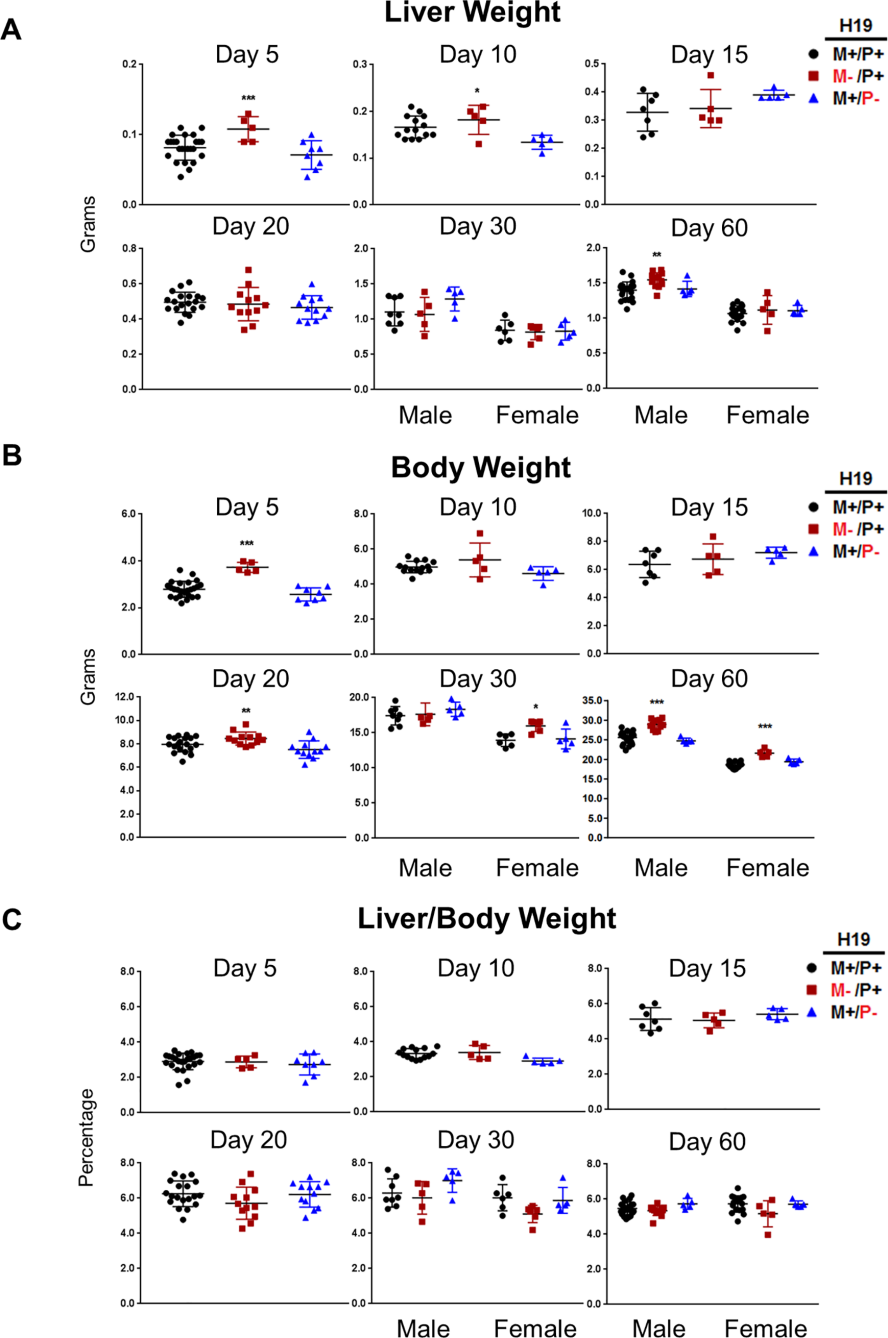
Liver and body weights for mice with *H19* knockout on different paternal alleles. Liver weights (A), body weights (B), and liver weight as a percentage of total body weight (C) were measured at the time of liver harvest from mice ages 5, 10, 15, 20, 30, and 60 days after birth (n = 5–25). Values are represented as mean ± S.D. *p<0.05, **p <0.01, ***p<0.001.

H19 impacts liver weight in the developing and adult male livers (Fig 4A). Liver weights are significantly higher immediately after birth and in male adult mice not expressing H19. Liver weights were significantly higher 5 days (p<0.001) and 10 days (*p*<0.05) after birth in mice without H19 expression. No significant changes in liver weight were observed between wild type mice and mice not expressing H19 for ages 15, 20, or 30 days after birth. Adult males, but not adult females, have significantly higher (*p*<0.01) liver weights when measured 60 days after birth with *H19* knockout on the maternal allele (*H19*^M-/P+^).

H19 impacts total body weight in developing and adult mice in both males and females (Fig 4B). Despite H19 expression not significantly affecting liver weight in adolescent or adult female liver, body weights are significantly altered at both 30 (*p*<0.05) and 60 (*p*<0.001) days after birth. Liver/body weight ratios were largely unaffected indicating weight changes were not specific to liver (Fig 4C). H19 also controls body weights when the liver is developing. Body weights are significantly higher with no H19 expression at 5 days after birth (*p*<0.001) and 20 days after birth (*p*<0.001). Both males and females had significant (*p*<0.001) increases in body weight at 60 days after birth when H19 was not expressed. Females, but not males, were significantly heavier at 30 days after birth (*p*<0.01) with no H19 expression through life.

### Changes in liver cell proliferation in the absence of H19 expression during postnatal maturation

**Fig 5:**
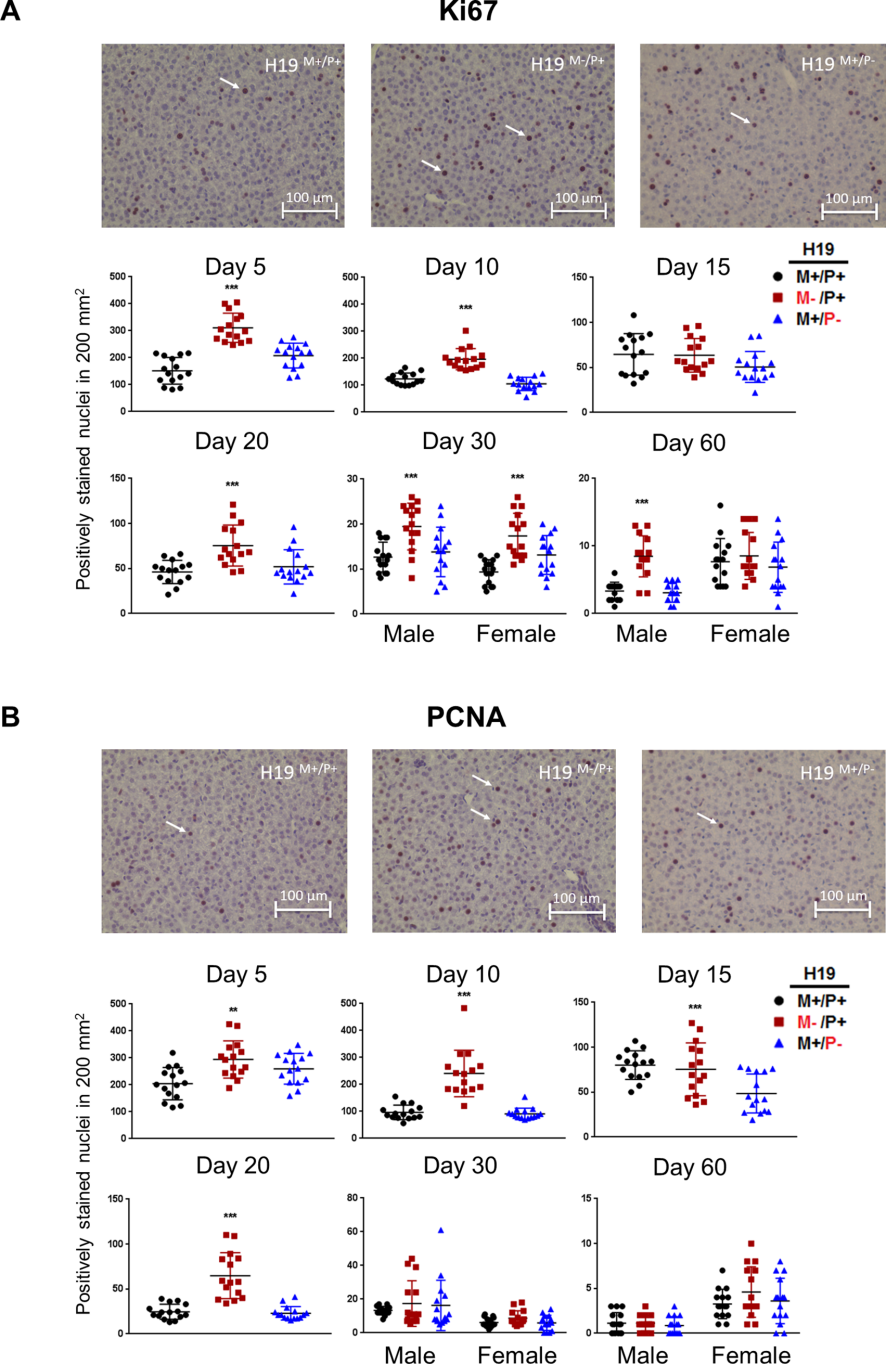
Immunohistochemistry analysis for identification of proliferating cells in livers of mice with *H19* knockout on different paternal alleles. Livers were fixed in formalin, embedded in paraffin, sectioned, and stained for (A) Ki67 or (B) PCNA for mice at ages 5, 10, 15, 20, 30, and 60 days after birth (n = 3 per group). A representative image was taken from mice at age 20 days after birth. Arrows indicate cells stained positive for (A) Ki67 and (B) PCNA. ImageJ software was used to count five sections per mouse. Values are represented as mean ± S.D. **p <0.01, ***p<0.001.

Corresponding data indicates liver weights are increased due to increases in cell proliferation throughout mouse development when H19 is not expressed. Two markers of cell proliferation, Ki-67 and PCNA, were used to measure proliferating cells in the tissue sections (Fig 5). When stained for Ki-67, livers from *H19* maternal allele knockouts (*H19*^M-/P+^) show significantly more Ki-67 positive nuclei in livers of mice at ages 5 (*p*<0.001), 10 (*p*<0.001), 20 (*p*<0.001), 30 in both males and females (*p*<0.001), and 60 only in males (*p*<0.001) days after birth. PCNA was also used to measure cell proliferation in an independent experiment. These data closely resemble Ki-67 staining results, indicating livers without H19 expression proliferate more rapidly during development when compared to wild type. In the maternal allele knockout mice, there were significantly more cells stained positive for PCNA at ages 5 (*p*<0.01), 10 (*p*<0.001), 15 (*p*<0.001), and 20 (*p*<0.001) days after birth. Similar to Ki-67 results, significant differences were observed throughout postnatal liver development using PCNA staining. Significant cell proliferation was only observed utilizing Ki-67 staining at later ages 30 and 60 days after birth indicating significant changes for *H19* maternal allele knockout mice (*H19*^M-/P+^) while PCNA did not stain significantly different at either of these ages. Measurements for heterozygous paternal allele *H19* mutants (*H19*^M+/P-^) resemble wild type (*H19*^+/+^).

### Expression of IGF signaling and Wnt signaling genes in the absence of H19 expression during postnatal maturation

**Fig 6:**
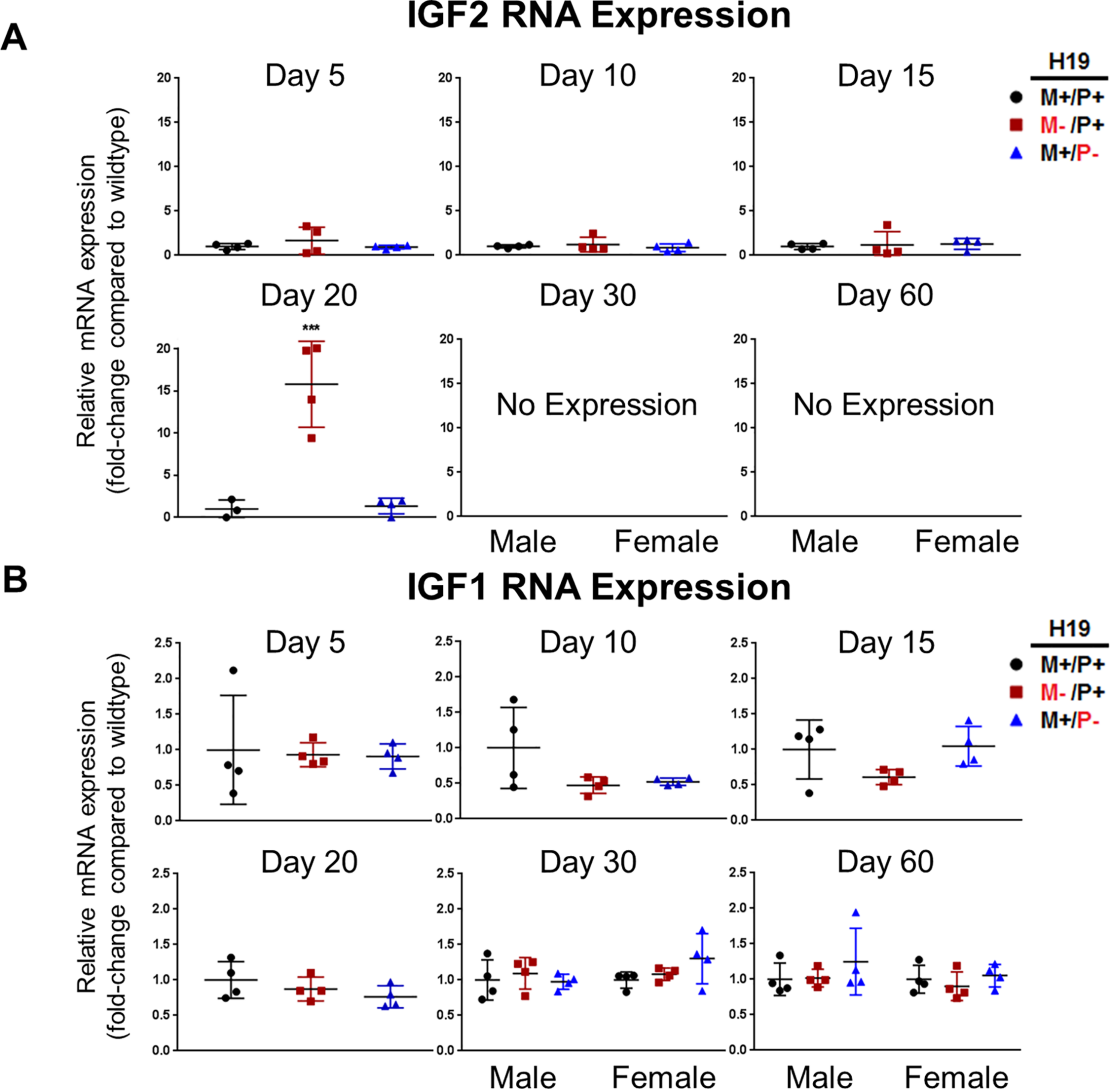
Expression of the fetal form IGF2 and adult form IGF1 RNA in mouse liver with *H19* knockout on different parental alleles. RNA expression was determined by RT-PCR for (A) IGF2 and (B) IGF1 in mouse livers at ages 5, 10, 15, 20, 30, and 60 days after birth (n = 4 per group). Values are represented as mean ± S.D. ***p<0.001.

Gene expression patterns for IGF2 and IGF1 were determined using RT-PCR for mice at ages 5, 10, 15, 20, 30, and 60 days after birth (Fig 6A and 6B, respectively). Both H19 and IGF2 were highly expressed at early ages until 20 days after birth when expression precipitously declines to undetectable levels and no expression persists through adult life. IGF1 exhibits the opposite expression pattern, starting with low expression early in life, steadily increasing as the liver develops. A significant increase (*p*<0.01) in IGF2 mRNA was found in *H19* maternal allele knockout mice (*H19*^M-/P+^) compared to wild type (*H19*^+/+^) at 20 days after birth, indicating H19 affects IGF2 expression. However, at all other ages measured, no significant differences were found. IGF1 mRNA was not impacted by the absence of H19 expression.

**Fig 7:**
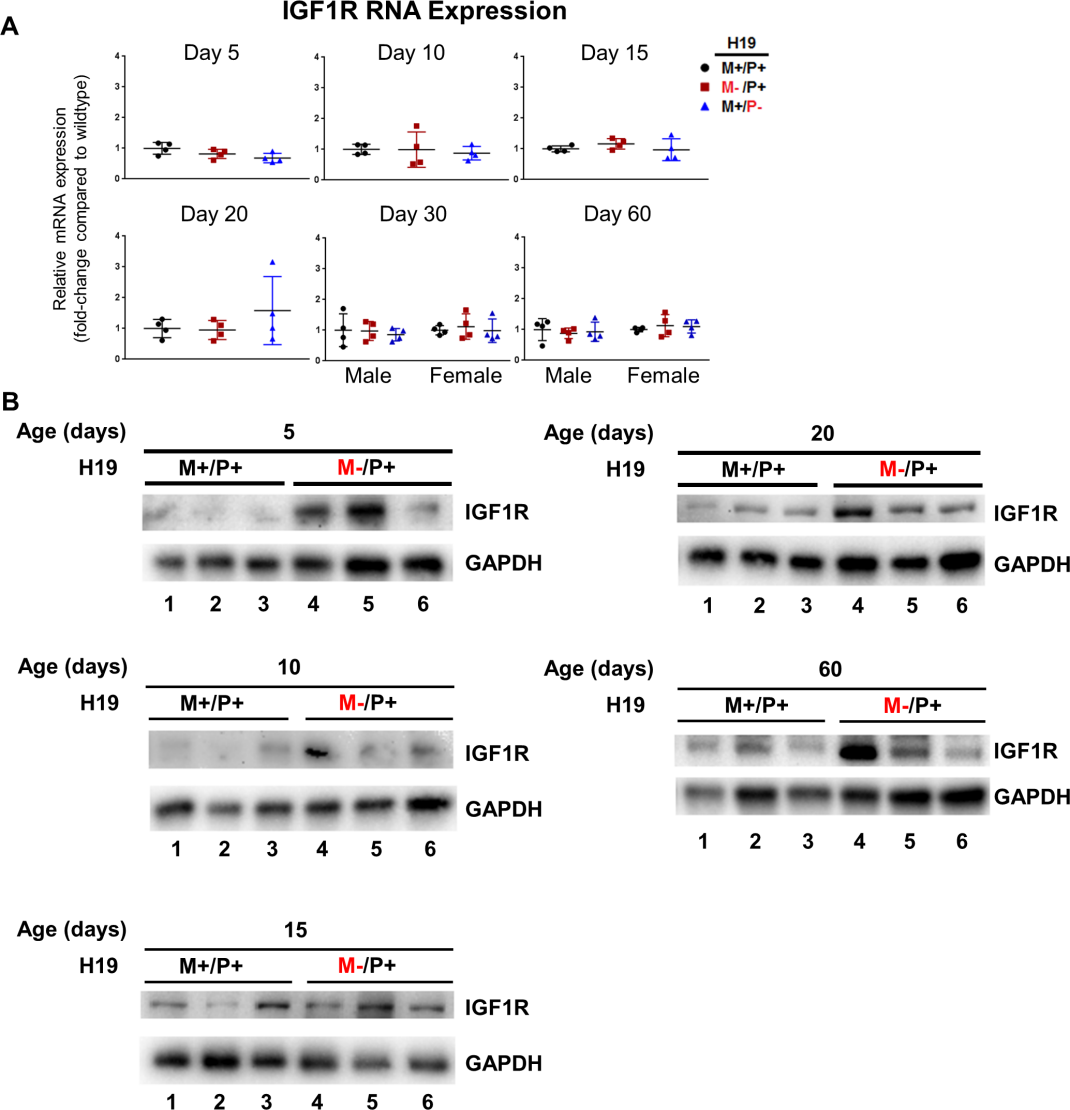
Expression of IGF1R in mouse livers with *H19* knockout on different parental alleles. (A) RNA expression was determined by RT-PCR in mouse livers at ages 5, 10, 15, 20, 30, and 60 days after birth (n = 4 per group). (B) Protein expression was determined by Western blot for IGF1R for mice at ages 5, 10, 15, 20, and 60 days after birth. Lanes 1–3 indicate wild type (*H19*^+/+^) individuals and lanes 4–6 indicate *H19*^M-/P+^ individuals. Values are represented as mean ± S.D.

IGF1R expression was found to increase only at the protein level, but not at the mRNA level in *H19* maternal allele knockouts (*H19*^M-/P+^) compared to wild type (*H19*^+/+^). Although no significant differences were observed in mRNA expression at all ages measured (Fig 7A), there was an increase in IGF1R protein expression when H19 was not expressed for each time point measured (Fig 7B). Levels of IGF1R protein expression were highly variable between individuals at each age and experimental groups.

**Fig 8:**
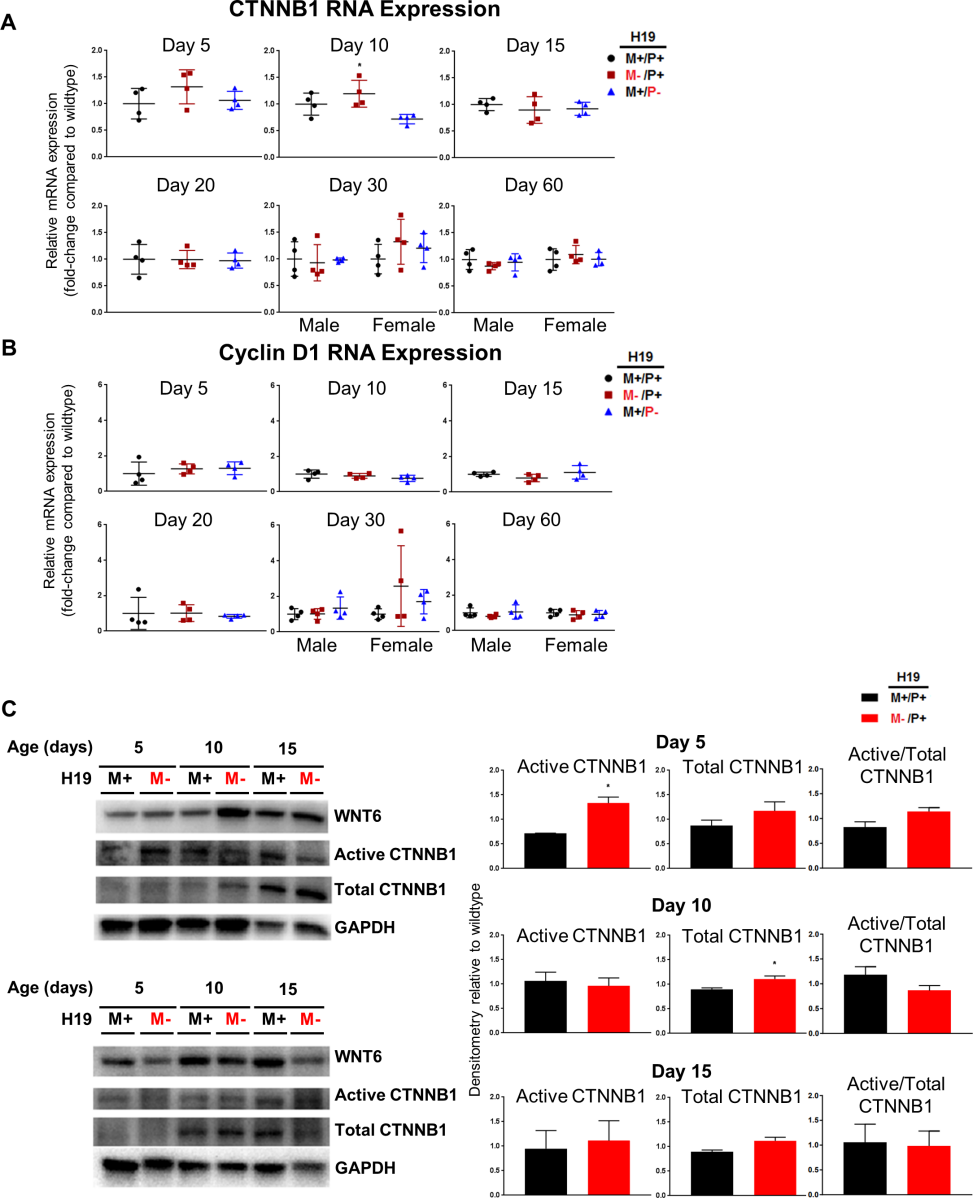
Expression of Wnt signaling in mouse livers with *H19* knockout on different parental alleles. RNA expression was determined by RT-PCR for (A) β-catenin and (B) cyclin D1 in mouse livers at ages 5, 10, 15, 20, 30, and 60 days after birth (n = 4 per group). (C) Protein expression was determined by western blot for Wnt6, active β-catenin, and total β-catenin for mice at ages 5, 10, and 15 days after birth (n = 2 per group). Values are represented as mean ± S.D. *p<0.05.

Wnt signaling was similarly only slightly impacted by the absence of H19 in mice. Only a significant increase (*p*<0.05) in β-catenin was observed at 10 days after birth at the mRNA level, and no changes were observed for cyclin D1 (Fig 8A and 8B, respectively). A significant increase (*p*<0.05) was observed at the protein level for active β-catenin in mice at age 5 days after birth. Total β-catenin in mice at age 10 days after birth was also significantly increased (*p*<0.05), and this measurement coincides with the increase observed at the RNA level. Despite these changes, no difference in active/total β-catenin was observed (Fig 8C). Protein expression was also analyzed for the ligand Wnt6, but no changes were observed.

### Expression of P450 drug metabolizing enzymes in the absence of H19 expression during postnatal maturation

**Fig 9:**
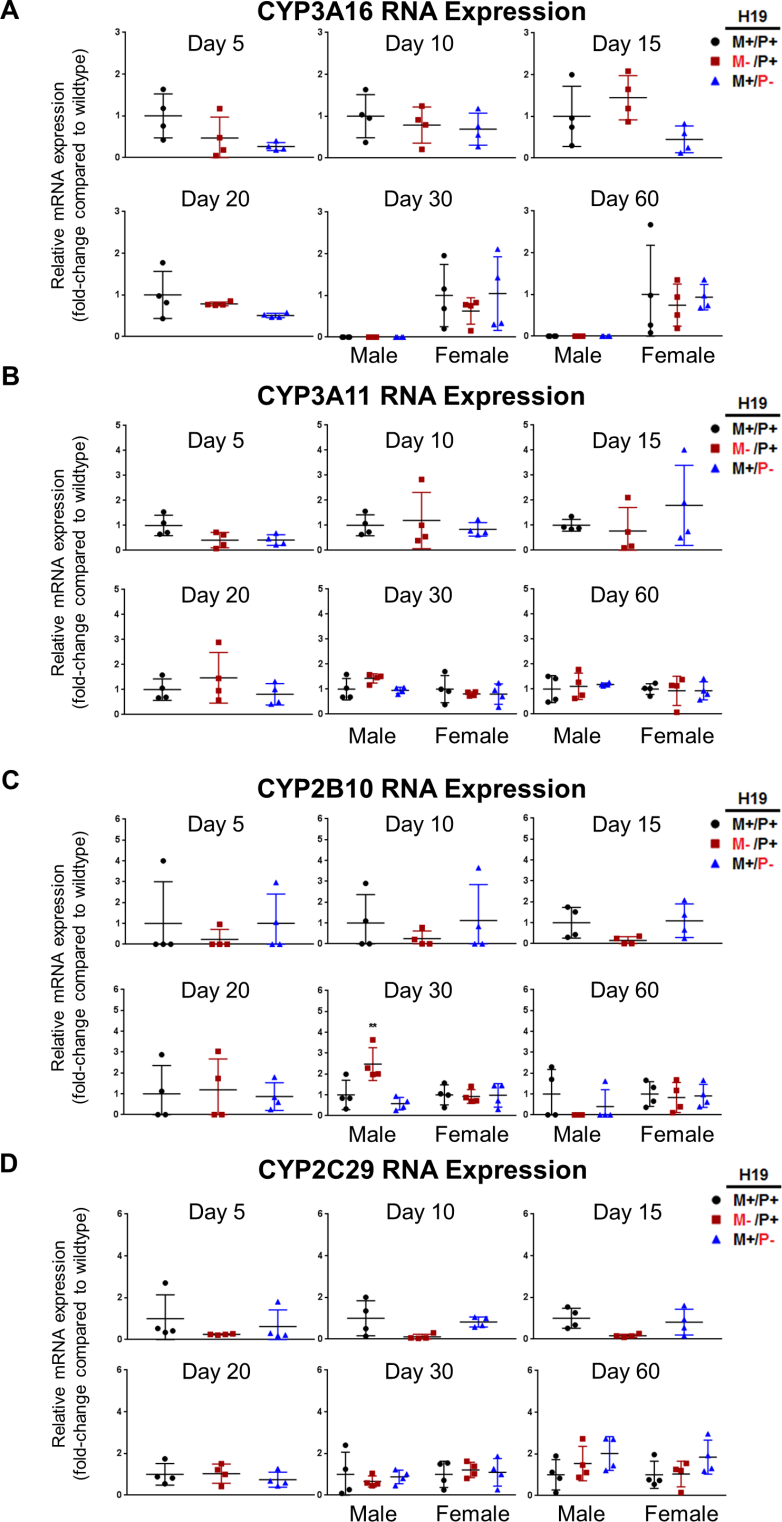
Expression of P450 drug metabolizing enzymes in mouse livers with *H19* knockout on different parental alleles. RNA expression was determined by RT-PCR for (A) CYP3A16, (B) CYP3A11, (C) CYP2B10, and (D) CYP2C29 RNA in mouse livers at ages 5, 10, 15, 20, 30, and 60 days after birth (n = 4 per group). Values are represented as mean ± S.D. **p <0.01.

Gene expression was determined for P450 enzymes in *H19* paternal allele knockouts (*H19*^M+/P-^), *H19* maternal allele knockouts (*H19*^M-/P+^), and wild type mice (*H19*^+/+^). There were no significant differences in mRNA expression for CYP3A16 (Fig 9A), CYP3A11 (Fig 9B), or CYP2C29 (Fig 9D). A significant increase (*p*<0.01) was found in CYP2B10 in *H19* maternal allele mutants (*H19*^M-/P+^) compared to wild type (*H19*^+/+^) for male mice aged 30 days (Fig 9C). Significance was not found in females aged 30 days or at any other ages measured for CYP2B10. CYP2C29 expression was delayed in mice when H19 expression is not present early in life (Fig 9D). Normally, as seen in wild type (*H19*^+/+^) and in paternal *H19* allele knockouts (*H19*^M+/P-^), CYP2C29 begins to be slightly expressed at 15 days after birth. However, when H19 is not expressed (*H19*^M-/P+^), CYP2C29 is not expressed until later in development and resumes its full normal expression at 20 days after birth.

### Expression of α-fetoprotein and albumin protein in the absence of H19 expression during postnatal maturation

**Fig 10:**
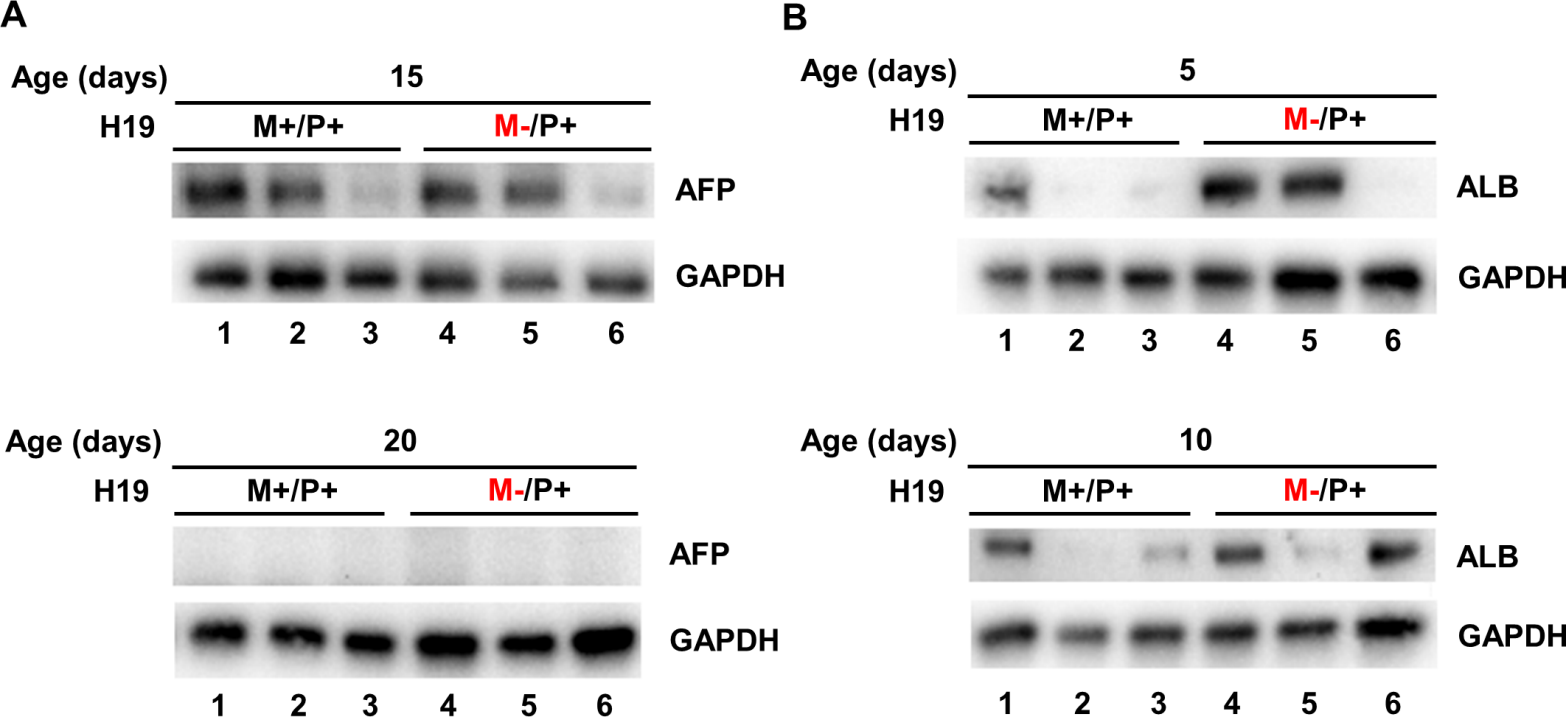
Protein expression of α-fetoprotein and albumin in mouse livers with *H19* knockout on the maternal allele. Protein expression was determined by Western blot for (A) α-fetoprotein in mouse livers ages 15 and 20 days after birth, and (B) albumin in mouse livers ages 5 and 10 days after birth. Lanes 1–3 indicate wild type (*H19*^+/+^) individuals and lanes 4–6 indicate *H19*^M-/P+^ individuals. Values are represented as mean ± S.D.

Liver postnatal protein expression for α-fetoprotein was examined in mice at ages corresponding to the decline of expression of H19 in normal wild type mice. No significant differences in α-fetoprotein were observed between *H19* maternal allele knockouts (*H19*^M-/P+^) and wild type (*H19*^+/+^) (Fig 10A). In mice aged 20 days after birth, no α-fetoprotein protein expression was found in liver for either test group. As observed in IGF1R protein expression, large interindividual differences were found between the three mice tested in each group.

Liver postnatal protein expression for albumin was examined in mice at the early ages of development. Western blots indicate no significant difference in protein expression of albumin in livers of mice at ages 5 or 10 days after birth (Fig 10B) for either test group. As with α-fetoprotein, there were large interindividual differences.

## Discussion

Research on H19 in normal liver development has primarily focused on fetal development with little regard to H19’s role after birth despite this being a time when the liver is continually growing and is dynamically changing its function from being a hematopoietic organ to a metabolic organ. When discovered, Pachnis *et al*. initially characterized H19 expression in mouse from before birth through the postnatal age until expression is terminated, by their observation, around 28 days after birth [17]. We have recapitulated their initial temporal expression pattern using modern techniques and expanded upon their seminal observations with a focus on H19’s role in postnatal liver maturation, including both liver growth and changes in the liver’s ability to achieve adult liver functions.

Liver growth is accelerated early in postnatal liver development when H19 is not expressed. The overgrowth phenotype has been previously characterized for mice inheriting the *H19*-null allele from their mother [25]. Heterozygous mice used in this study containing a functional maternal allele but with a mutated paternal allele resemble homozygous wild type mice throughout our observations, as the paternal allele was inconsequential to H19 expression. We observed the overgrowth phenotype by measuring both the total body and liver weights through the postnatal ages. Early in life, liver weights are significantly affected when H19 is not expressed (Fig 4A). Coordinately, we also measured the level of cell proliferation in liver and found significant changes in positively stained nuclei for two different cell proliferation markers, indicating enhanced proliferation that persists through many measured postnatal ages (Fig 5). Ki-67 is preferentially expressed during late G_1_, S, G_2_, and M phases of the cell cycle, but not in the resting G_0_ phase [26]. PCNA is an accessory protein for DNA polymerase alpha required for DNA synthesis, and is elevated during the G1/S phase of the cell cycle [27]. Both markers indicate that the absence of H19 expression enhances cell proliferation in the developing mouse livers.

Increases in cell proliferation do not directly result in increases in liver mass. There is a discordance with observations in significant liver weight increases and increases in the markers indicating cell proliferation in *H19* maternal allele knockout mice. Liver weights are only significantly increased immediately in early life and in adult males while increases in proliferation are observed throughout most of liver development at postnatal ages. Interestingly, Ki-67 staining indicates no significant increases in cell proliferation at 15 days after birth despite showing significant increases before and after this age in the absence of H19 expression, suggesting H19 does not influence proliferation at this specific time of development, or changes were only detectable when assaying for PCNA expression.

Loss-of-function of *H19* in developing mice induces changes in liver that persist even at ages when H19 is not normally expressed (ages 30 and 60 days after birth). An increase in adult male liver weight can be explained by the accumulation of organ mass at earlier ages too subtle to have been detected at prior ages. Increases in proliferation as observed by Ki-67 at ages 30 and 60 days after birth in *H19* maternal allele knockout mice may be the result of more complex pathway changes that are initiated at the time of normal H19 expression and persist after H19 is no longer expressed. These late age changes were not observed in the PCNA stain (Fig 5B).

We then sought to determine how the loss of H19 expression results in an increase in liver mass and an increase in proliferating cells throughout postnatal development by first examining IGF signaling. There is a strong correlation in expression patterns between lncRNAs and protein coding genes within the same loci, suggesting *cis* regulation with the lncRNA potentially influencing its protein coding partner [15]. Others have shown that regulation is not always the lncRNA acting directly on the protein coding gene, but rather its promoter or nearby regulatory regions that influence gene expression of nearby genes [28]. *H19* and *Igf2* reside next to each other on chromosome 7 in the mouse and have similar temporal expression patterns in postnatal liver. Different *H19*-null mouse models have been used to determine the function of not only H19 RNA, but also regulatory sequences surrounding its locus. Some mouse models have deletions that span into the *Imprinting Control Region (ICR)* between *H19* and *Igf2* resulting in disruption of imprinting of *Igf2*, which authors have concluded leads to biallelic expression of IGF2 and the overgrowth phenotype [25]. However, our model has only *H19* and a portion of its promoter containing an Sp1 site and TATA box removed, leaving the *ICR* intact allowing us to study only the effects of H19 on postnatal liver development [22]. Our results demonstrate that deletion of the *ICR*, causing bialleleic expression of IGF2, is not needed to induce the overgrowth phenotype.

Our measurements indicate H19 has a minimal effect on IGF2 expression, with significant increases in IGF2 expression observed only at 20 days after birth (Fig 6A). However, at 5 days after birth, the liver overgrowth phenotype is observed (Fig 4A) and cellular proliferation is significantly increased (Fig 5) throughout most postnatal ages when H19 is not expressed. This indicates other pathways may be affected by the *H19* loss-of-function.

The Wnt signaling pathway has been shown to be important in the development of many different tissue types and organs. The canonical intracellular transducer, β-catenin, is activated after the Wnt ligand binds to a Frizzled family receptor. This activation causes β-catenin accumulation in the cytoplasm and its eventual localization into the nucleus where it acts as a coactivator of transcription factors, affecting gene transcription [29]. Cyclin D1, a cell cycle inducer important for the G_1_ to S phase transition, is a target for regulation by β-catenin [30] in many different physiological processes including liver growth [31]. Wnt signaling has been shown to be inhibited by H19 in fetal liver leading to inhibition of cell proliferation [32], and Wnt signaling influences proliferation in liver at postnatal ages [11]. A significant increase was observed at the mRNA level for β-catenin for mice age 5 days after birth, however, no changes were observed for a downstream gene target of the pathway important for cellular proliferation, cyclin D1, when H19 is not expressed (Fig 8). Protein expression was also analyzed for the Wnt ligand and both activated β-catenin and total β-catenin. Antibody detection against Wnt6 was chosen due to Wnt6 being involved in canonical signaling and its high expression in developing tissues [33]. No significant changes were observed in Wnt6 expression, indicating Wnt signaling was affected only downstream in the pathway or changes occurred in other Wnts not examined. Significant differences were discovered at the protein level in early life (age 5 days after birth) for active β-catenin, and total β-catenin is also significantly higher at 10 days after birth indicating similar results as observed in prior studies examining fetal H19 inhibition of β-catenin protein [32]. It is not surprising many significant changes were not observed at the RNA level for genes within the canonical pathway. However, mRNA expression for cyclin D1, which is a direct target of Wnt signaling at the transcriptional level, was not found to be significantly altered.

H19 encodes a microRNA, miR-675, within its first exon [34]. *H19* knockout on the maternal allele abolishes expression of both H19 and miR-675 despite the status of the paternal allele (Fig 3). H19 potentially impacts liver growth and proliferation through the action of miR-675. Prior literature has shown proper processing of miR-675 can slow growth in the placenta, and increases of miR-675 downregulate IGF1R, which causes IGF signaling to be inhibited [35]. Consistent with our data, removal of H19 expression only causes a significant increase (Fig 6A) in IGF2 at 20 days after birth despite detection of increased liver weights and cell proliferation at earlier ages. This suggests involvement of another regulator. Inhibition of IGF1R by miR-675 may be the mechanism by which *H19* controls liver growth. IGF1R expression was examined at both the RNA and the protein levels. No significant differences were found, but a trend of an increase in IGF1R expression at the protein level was found for each age measured, suggesting a loss of miR-675 expression may be the cause for the overgrowth phenotype and the increase in cell proliferation.

The developmental P450 expression pattern for Cyp3a is not influenced by H19 expression. The CYP3A family undergoes a switch in dominant isoforms during postnatal liver development. Early in life, CYP3A16 is the dominant isoform in mice. The adult CYP3A isoform switches to predominantly CYP3A11 around 20 days after birth [36]. Despite this developmental shift pointing to CYP3A potentially being impacted by H19 expression, no significant differences were observed between wild type mice and mice with the maternal *H19* allele knocked out (Fig 9A and 9B).

H19 does appear to affect the expression of P450 enzymes CYP2B10 and CYP2C29 at particular points during mouse liver development. Sex differences in CYP2B10 expression are known between wild type males and females. Wild type adult females display a higher expression level than adult males in mouse liver [37]. Our results also point to a sex difference in H19’s role affecting the expression of CYP2B10 only significantly in male mice and not female mice at 30 days after birth (Fig 9C). CYP2C29 is not normally expressed until the liver begins to mature. In wild type mice, slight expression is observed at 15 days after birth. In *H19* maternal allele knockouts with no H19 expression, the expression of CYP2C29 only begins to be detected at 20 days after birth, and at 15 days after birth is not expressed (Fig 9D). Despite these specific changes, P450s were largely found to be unaffected by H19 expression indicating H19 may not be important for liver maturation.

Insignificant changes in expression patterns of albumin and α-fetoprotein, two developmentally regulated genes, also indicate H19 may not be important for liver maturation. Albumin production is a function and marker of normal mature hepatocytes, but can be detected in nascent hepatic cells [38]. Albumin production rises continually throughout liver development and is at maximum in adult liver [39]. Due to this expression pattern, we chose the two earliest ages in our study (5 and 10 days after birth) to examine changes in albumin production in developing liver in mice with and without H19 expression. If H19 affects the maturation of liver, a difference in levels of albumin production might be noticed at different developmental ages. Conversely, α-fetoprotein is a fetal liver gene and the major plasma protein present in the fetus. The RNA expression profile of α-fetoprotein in postnatal liver development resembles H19, with highest expression early in life and a dramatic decline after birth. Expression of α-fetoprotein mRNA declines to undetectable levels around 14 days after birth in mouse [17]. Due to this pattern, we chose to examine protein expression of α-fetoprotein at ages 15 and 20 days after birth between wild type mice and mice not expressing H19. Protein expression was still detected at 15 days after birth in both groups, but by 20 days after birth, α-fetoprotein protein was not observed for either test groups (Fig 10A). No significant changes were observed in the production of albumin or α-fetoprotein when H19 is not expressed (Fig 10) indicating H19 may not control these developmentally regulated genes.

H19’s role in postnatal liver maturation appears to be consistent with its role in other contexts. H19 is expressed in highly proliferating tissues including fetal and postnatal livers [17], and H19 expression can reemerge in adult liver during hepatocellular carcinoma [19,20], or during regeneration after injury [17,40]. Despite its expression in proliferating tissue, our data support the hypothesis that normal expression or reemergence of H19 is to limit cellular proliferation to control overgrowth.

## Conclusions

H19 affects liver growth controlling proliferation through IGF and Wnt signaling, but may be inconsequential to liver maturation during postnatal development. H19’s action is potentially through miR-675. miR-675 has been shown to inhibit IGF1R indicating uninhibited IGF signaling may be the cause of the overgrowth phenotype and increases in cell proliferation. Despite H19’s regulation of liver growth, evidence suggests H19 may not play a significant role in postnatal liver maturation. Albumin and α-fetoprotein expression patterns were not significantly altered, and P450 expression pattern changes were only affected at specific ages.

## Supporting information

S1 Minimal Data Set (XLSX)
